# Linking Ghrelin Elevation to Reproductive Dysfunction: Insights from Feed-Restricted Male Rats

**DOI:** 10.1007/s43032-025-01943-2

**Published:** 2025-08-12

**Authors:** Marina M. Ibrahim, E. Seifelnasr, Marwa A. Ibrahim, Eman I. Hassanen, Asmaa S. Morsi, Manal R. Bakeer

**Affiliations:** 1 Department of Physiology, Faculty of Veterinary Medicine, Cairo University, Cairo, Egypt; 2 Department of Biochemistry and Molecular Biology, Faculty of Veterinary Medicine, Cairo University, Cairo, Egypt; 3 Department of Pathology, Faculty of Veterinary Medicine, Cairo University, Cairo, Egypt

**Keywords:** Ghrelin, Feed restriction, Hypothalamic-pituitary–gonadal axis, Fertility, Adult male rats

## Abstract

Ghrelin, a peptide hormone consisting of 28 amino acids and primarily produced in the stomach, is crucial for stimulating appetite and maintaining energy balance. Beyond its role in hunger and metabolism, ghrelin also influences the hypothalamic-pituitary–gonadal (HPG) axis, impacting reproductive hormones secretion. During starvation, ghrelin levels increase as part of the body's energy conservation response. The study explored the impact of feed restriction on ghrelin levels and the expression of the ghrelin gene in the gastric fundus, hypothalamus, and testes, as well as their association with reproductive function in adult male rats. Thirty adult male Swiss albino rats were assigned to three groups: a control group (ad libitum feeding), a 30% feed restriction group, and a 60% feed restriction group, over two months. An additional ten adult female rats were included to assess changes in male sexual behavior. Feed restriction resulted in a marked increase in ghrelin levels and its gene expression in the examined tissues, with the most pronounced elevation observed in the 60% feed restricted group. Elevated ghrelin disrupted the HPG axis, leading to reduced levels of follicle-stimulating hormone (FSH), luteinizing hormone (LH), and testosterone, while prolactin levels increased. These hormonal changes negatively impacted fertility, libido, semen quality, and overall sexual function. Additionally, feed restrictions caused histopathological alterations in the stomach and testes, along with reductions in both testicular and body weight. These findings suggest that increased ghrelin hormone, in response to energy deficiency, may inhibit the male reproductive axis, linking energy balance and reproductive function in male rats.

## Introduction

Ghrelin is a hormone mainly produced by the stomach, playing a key role in regulating appetite and ensuring energy balance [1]. Ghrelin exists in two main forms: acylated ghrelin, which is biologically active and responsible for stimulating appetite, and des-acyl ghrelin, which is devoid of the acyl group and does not possess the same appetite-stimulating effects [2]. The balance between these two forms and their respective plasma levels is crucial for maintaining energy homeostasis and regulating appetite [3].

Plasma ghrelin levels fluctuate based on various physiological conditions, including dietary intake, energy status and body weight [4]. Typically, ghrelin concentrations increase during fasting, signaling hunger to the brain, and decrease following a meal [5].

The action of ghrelin occurs through the growth hormone secretagogue receptor (GHSR), which stimulates appetite and reduces energy expenditure [6]. Ghrelin also has broader physiological functions, including the regulation of cardiovascular function, promotion of bone growth, support of gastrointestinal processes, and influence on neurogenesis and muscle formation [7].

Ghrelin and the growth hormone secretagogue receptor (GHSR), are found in various organs and reproductive tissues throughout the body [8]. Such receptors are present in the hypothalamus and pituitary gland, which are critical for regulating hormonal balance and reproductive functions [9]. Additionally, GHSRs are located in the ovaries, testes, placenta, and other reproductive tissues, highlighting the potential influence of ghrelin on reproductive physiology [10].

Like many metabolic hormones, ghrelin is a pleiotropic hormone with diverse functions, including a proposed regulatory role in the reproductive axis [11]. It plays a key role in connecting energy balance to reproductive health by regulating the hypothalamic-pituitary–gonadal (HPG) axis, emphasizing the importance of energy homeostasis in maintaining optimal reproductive function [12].

The objective of this research is to assess the impact of chronic feed restriction at two varying levels of severity on ghrelin hormone concentrations and to assess how these alterations affect reproductive hormones and sexual function in adult male rats.

## Materials and Methods

### Ethical Approval

The study was carried out in the Physiology Department of the Faculty of Veterinary Medicine at Cairo University, after approval of the experimental procedures by the Institutional Animal Care and Use Committee of the Faculty of Veterinary Medicine, Cairo University. (VET.CU.IACUC, approval number Vet Cu131020241054). The rats had a two-week period to acclimate to the laboratory environment prior to the start of the experiment. During this period, they were fed a standard granulated diet containing 18% crude protein, 86% dry matter, 5.5% fiber, 2.5% fat, and essential minerals and vitamins, with ad libitum access to water. The rats were housed in a separate room with natural ventilation, under a 12-h light/dark cycle, at a temperature of 20–24 °C, and humidity levels ranging from 60–65%. They were randomly assigned to groups and accommodated in plastic cages (35.60 × 63.55 × 24.68 cm) equipped with galvanized iron filter tops.

### Animals and Experimental Design

The experiment involved 30 adult male Swiss rats with body weights ranging from 160 to 180 g. Additionally, 10 adult female rats, with weights ranging from 120 to 140 g, were included to evaluate the sexual behavior of the male rats at the end of the experiment. All rats were procured from the Medical Entomology Research Institute in Giza, Egypt.

The male rats were assigned to three groups (*n = *10):

- Group 1 (control group): Fed ad libitum with a standard rat diet.
- Group 2 (30% feed-restricted group): were provided with 70% of the ad libitum intake.
- Group 3 (60% feed-restricted group): were provided with 40% of the control group's feed intake.

The male rats were housed in individual cages to eliminate any influence of social hierarchy or competition over food, ensuring that each animal received the exact amount of feed allocated according to its group. This approach allowed for precise monitoring and control of food intake per animal and minimized variability due to unequal access to food. The feed consumption for Groups 2 and 3 was determined based on the amount consumed by the control group from the beginning of the experiment until its end [13]. All groups were provided with unrestricted access to water throughout the experiment, which lasted for two months.

The rats were weighed weekly throughout the study to reduce the impact of recent feed and water intake on body weight measurements. Before weighing, all rats were fasted and deprived of water for 12 h. After the experiment, the rats were weighed again to determine their final body weights.

At the end of the study, estrous synchronization was conducted for the ten adult female rats to evaluate the sexual behavior of the adult male rats. This synchronization involved administering two intramuscular injections of estrumate (15 µg per rat) three days apart [14]. A vaginal smear was performed to detect whether the females were in the estrus phase. The female rats were assessed for receptivity prior to being introduced to the males. Those that exhibited lordosis after being mounted by a male rat were included in the study of sexual behavior [15].

### Evaluation of Sexual Behaviors

In a quiet room maintained at a temperature of 23 °C to 26 °C and a relative humidity of 60% to 65%, sexual behavior testing was conducted on five randomly selected male rats from each group. Each male was individually placed in a mating cage (34.5 cm × 45.5 cm × 23.5 cm) for a 5-min acclimation period. A sexually receptive female was then introduced into the cage, and a 30-min adaptation period followed to facilitate interaction. Mating behavior was subsequently observed, and sexual parameters were recorded for each male. To minimize repeated exposure and potential behavioral bias, females were rotated among different males across separate sessions. After each mating session, the cage was thoroughly cleaned and disinfected to eliminate pheromonal cues that could affect subsequent trials. All interactions were videotaped for later behavioral analysis. Testing was conducted during the late photophase (15:00–19:00) [15].

The sexual behavior parameters of each tested male in all groups were assessed over a 2-h observation period. The recorded parameters included first contact time, number of contact attempts, mount frequency (MF), mount latency, and number of licks [16].

### Collection and Analysis of Samples

At the end of the two-month experimental period, five rats from each group were randomly selected for reproductive and histological evaluations to ensure representative sampling and minimize selection bias. This randomization process enhances the validity of group comparisons by reducing potential confounding variables. Prior to sample collection, the selected rats were fasted overnight. The following morning, fasting blood samples were obtained after administering anesthesia via intraperitoneal injection of ketamine (90 mg/kg) and xylazine (10 mg/kg) [17]. Blood was drawn using heparinized capillary tubes by puncturing the orbital sinus. The blood samples were collected without anticoagulant and allowed to clot, after which the serum was stored at −20 °C for hormone assays (Ghrelin, Follicle-stimulating hormone (FSH), Luteinizing hormone (LH), Prolactin, and Testosterone (T)) using a double antibody immunoassay with specific ELISA kits obtained from LEADER TRADE Company in Egypt. The following kit IDs were used: Ghrelin (SG-20440), LH (SG-20701), FSH (SG-20702), Prolactin (SG-20835), and Testosterone (SG-20703).

### Semen Analysis

After the collection of all blood samples, male rats were euthanized under anesthesia for collection of semen and tissue samples [17]. The testes were collected and weighed, Semen was obtained from the cauda epididymis using a technique similar to the one outlined by [18, 19] to measure sperm concentration, motility (%), viability (%), and sperm abnormalities (%). Sperm concentration was measured using an improved Neubauer hemocytometer. To evaluate sperm motility percentage, a drop of undiluted semen was applied to a slide and observed under a microscope at a magnification of 10x. For sperm viability evaluation, a drop of semen was applied to a slide, then a drop of eosin-nigrosin stain was added, thoroughly mixed, and air-dried. The sample was subsequently examined under a microscope to distinguish between live (viable) and dead (non-viable) spermatozoa cells [20].

### Ghrelin Gene Expression

The testis, fundus of the stomach, and hypothalamus were stored in Eppendorf tubes at −80 °C until gene expression analysis was conducted to assess the impact of feed restriction (FR) on ghrelin gene expression.

Total RNA was extracted from the stomach, hypothalamus, and testes using the RNeasy Mini Kit (Qiagen, Cat. No. 74104). The quality and concentration of the RNA were measured with a spectrophotometer, and samples with an A260/A280 ratio ranging from 1.8 to 2.0 were chosen [21].

For cDNA synthesis, 1 µg of total RNA was used, following the manufacturer's instructions with Thermo Scientifi kit (RevertAid First Strand cDNA). The reaction was incubated at 25 °C for 10 min, at 42 °C for 60 min, and terminated by heating at 85 °C for 5 min.

Quantitative real-time PCR (qRT-PRC) was performed using the specific primers for the ghrelin gene: forward primer: 5’-AGCATGCTCTGGATGGACAT- 3’; reverse primer: 5’-TGCTTGTCCTCTGTCCTCTG −3’ and B-actin as a housekeeping gene: forward primer: 5’-CCGCGAGTACAACCTTCTTG-3’; reverse primer 5’-CAGTTGGTGACAATGCCGTG- 3’ [22]. Each PCR reaction was set up in a 20 µL volume using SYBRTM Green PCR Master Mix (Thermo Scientific) in Applied Biosystem qRT-PRC, and run in duplicates with no-template controls. The cycling conditions included an initial denaturation at 95 °C for 3 min, followed by 40 cycles of 95 °C for 15 s and 60 °C for 30 s, with a subsequent melting curve analysis from 65 °C to 95 °C to confirm product specificity [23]. Data analysis was conducted using the ΔΔCt method to determine relative gene expression, normalizing the ghrelin expression to the B-actin gene [24].

### Histopathological Analysis

Specimens from the fundus of the stomach and testes were collected from rats and preserved in 10% neutral buffered formalin. After 48 h, the specimens were processed through ascending grades of ethanol solutions, followed by xylene. The specimens were then embedded in paraffin wax and sectioned into 4.5 µm thick slices using a standard microtome. Subsequently, all sections were stained with Hematoxylin and Eosin (H&E) and examined under an Olympus light microscope to identify any abnormal histological structures. Finally, images were captured with an Olympus digital camera connected to CellSens Dimension software to be used for further statistical analysis [25]. Classical semiquantitative scoring was performed to assess the degree of lesion severity in each examined organ based on the lesion distribution in twenty microscopic fields per group. The four-pointed grading scale was used as follows: (0) normal histology, (+) mild changes, (+ +) moderate changes, (+ + +) severe changes.

### Statistical Analysis

Data analysis was performed using SPSS statistical software (version 11.0; SPSS Inc., Chicago, IL, USA). The results are expressed as the mean ± standard error of the mean (SEM). A one-way ANOVA was applied to evaluate significant differences between groups, with statistical significance defined as p < 0.05.

## Results

### The Impact of Feed Restriction on Ghrelin, FSH, LH, Prolactin and Testosterone in Male Rats

**Fig. 1:**
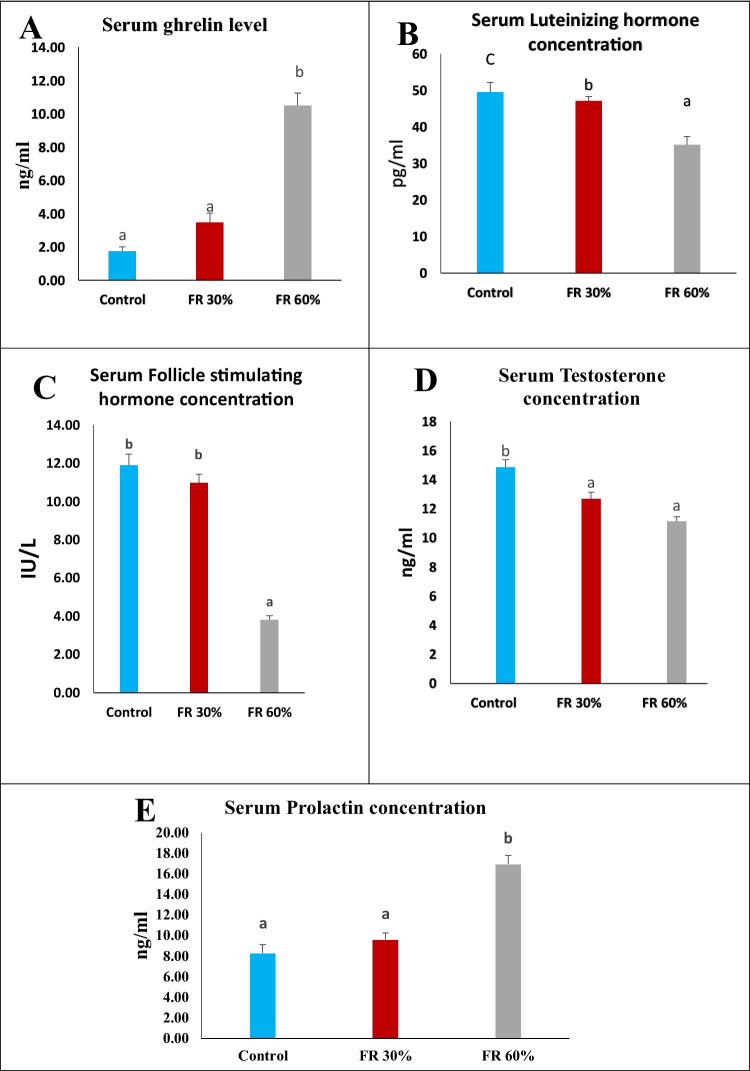
The impact of feed restriction on serum Ghrelin, LH, FSH, Testosterone and Prolactin levels in adult male rats. Data are presented as mean ± SEM (*n = *5 rats per group). Means with different letters (a, b, ab, c) indicate significant differences at *p* ≤ 0.05

The 60% feed-restricted group demonstrated a significant elevation (*p* < 0.05) in serum ghrelin and prolactin levels, while the 30% feed-restricted group exhibited no significant alteration compared to the control group, as shown in (Fig. 1A) and (Fig. 1E).

Data presented in (Fig. 1B), (Fig. 1C), and (Fig. 1D) reveal a significant reduction (*p* < 0.05) in testosterone, LH, and FSH levels in the 60% feed-restricted male rats when compared to the control group. Similarly, the 30% feed-restricted group exhibited significant reductions in testosterone and LH levels, but no significant alteration in FSH levels compared to the control group. Among the groups, the 60% feed-restricted group displayed the lowest levels.

### The Impact of Feed Restriction on Testicular Weight and Final Body Weight in Male Rats

**Fig. 2:**
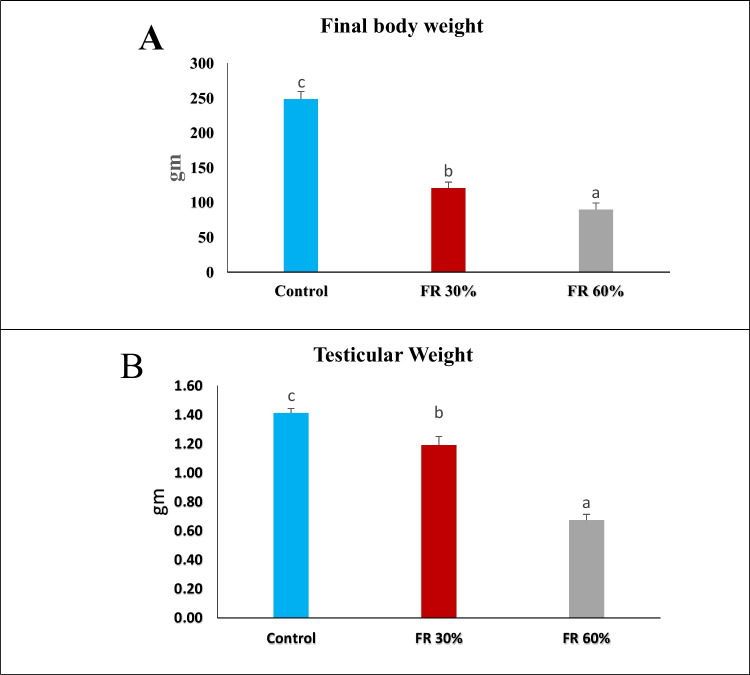
The impact of feed restriction on Final body weight and testicular weight in adult male rats. Data are presented as mean ± SEM (*n = *5 rats per group). Means with different letters (a, b, ab, c) indicate significant differences at *p* ≤ 0.05

The results presented in (Fig. 2A) and (Fig. 2B) showed a significant decrease in both testicular weight and final body weight in the 30% and 60% feed-restricted groups when compared to the control group.

### The Impact of Feed Restriction on Testis-Body Weight Ratio in Male Rats

The data presented in (Table 1) show that the 30% FR group exhibited a non-significant reduction in the testis-to-body weight ratio compared to the control group, whereas the 60% FR group showed a significant reduction.

**Table 1:** The impact of feed restriction on testis-body weight ratio (g/g) in male rats

Parameter	Testis-Body Weight Ratio (g/g)
Groups	
Control	0.0195 ± 0.0005^b^
30% FR	0.0188 ± 0.0006^ab^
60% FR	0.0125 ± 0.0004^a^

Data are presented as mean ± SEM (*n = *5 rats per group). Means with different letters (a, ab, c) indicate significant differences at *p* ≤ 0.05

### The Impact of Feed Restriction on Sexual Parameters in Male Rats

The data presented in (Table 2) indicate a significant increase (*p* < 0.05) in first contact time and mount latency time in Group 3 (60% FR) compared to Group 1 (control) and Group 2 (30% FR). In contrast, Groups 2 and 3 exhibited a significant decrease in the number of contact attempts, mount frequency, and licking frequency when compared to the control group. Furthermore, the 60% FR group exhibited a significant decrease in these parameters compared to the 30% FR group.

**Table 2:** The impact of feed restriction on sexual parameters in adult male rats

Parameters	First contact time (sec)	Contact number (attemp)	Mount frequency MF	Mount latency time(s)	Licking number
Groups					
Control	18.00 ± 0.87^a^	83.40 ± 4.35^c^	23.20 ± 1.35^c^	20.02 ± 0.53^a^	17.20 ± 0.96^c^
30% FR	29.94 ± 1.21^a^	54.60 ± 1.72^b^	16.20 ± 1.39^b^	35.48 ± 1.39^a^	12.80 ± 1.06^b^
60% FR	153.60 ± 5.31^b^	21.00 ± 1.58^a^	9.40 ± 0.81^a^	305.80 ± 10.64^b^	5.00 ± 0.70^a^

Data are presented as mean ± SEM (*n = *5 rats per group). Means with different letters (a, b, c) indicate significant differences at *p* ≤ 0.05

First contact time (sec) is the duration in seconds from the introduction of the female to the male's initial physical interaction or contact

Contact number (attempt) refers to the total count of attempts made to establish physical contact or initiate interaction during a given period

Mount Frequency (MF) refers to the total number of mounting attempts made by a male from the female's introduction to the point of ejaculation

Mount Latency (ML) is the duration between the female's introduction and the male's initial mounting attempt

Licking number refers to the total count of licking actions performed by a male rat on a female as part of courtship or mating behavior

### The Impact of Feed Restriction on Semen Analysis in Male Rats

**Fig. 3:**
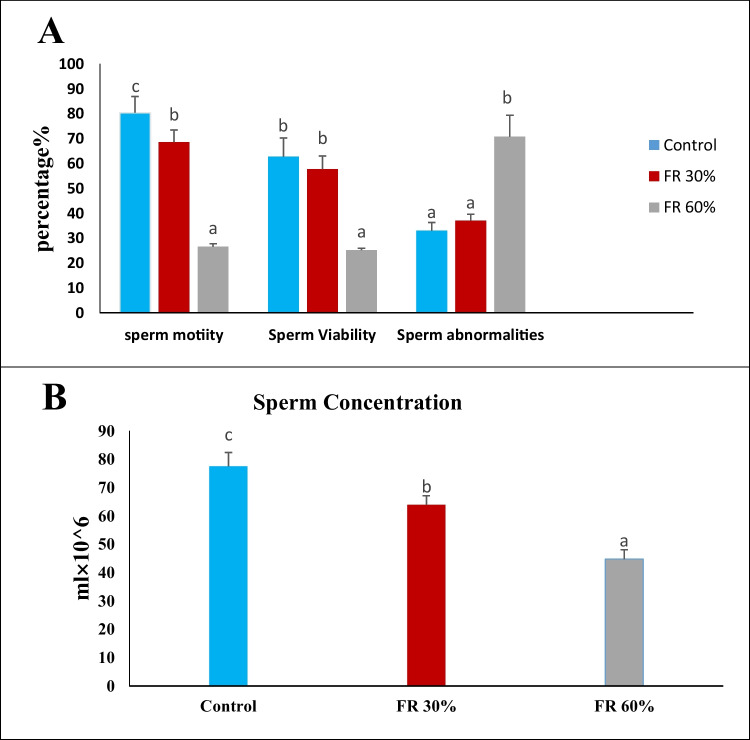
The impact of feed restriction on sperm motility, sperm viability, sperm abnormalities and sperm concentration in adult male rats. Data are presented as mean ± SEM (*n = *5 rats per group). Means with different letters (a, b, ab, c) indicate significant differences at p ≤ 0.05

The data shown in (Fig. 3A) and (Fig. 3B) demonstrate a significant reduction (*p* < 0.05) in sperm count, motility (%), and viability (%) in the feed-restricted groups (Group 2 and Group 3), with the 60% feed-restricted group displaying the lowest results. Additionally, a significant elevation (*p* < 0.05) in sperm abnormalities (%) was noted in the 60% feed-restricted group when compared to the control group.

### The Impact of Feed Restriction on the Expression of Ghrelin Gene in Male Rats

**Fig. 4:**
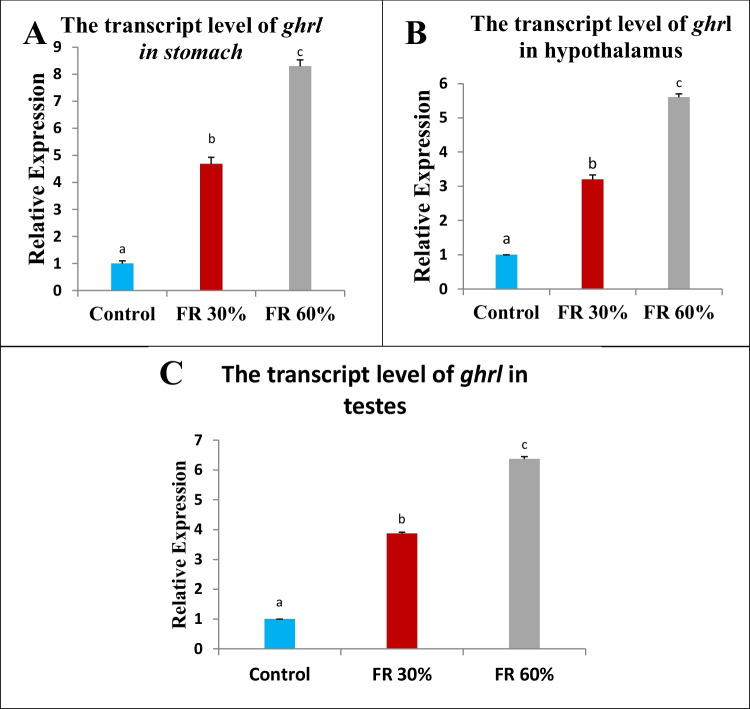
Bar chart representing the transcript levels of *ghrelin* gene among the different groups. Values are presented as mean ± SEM (*n = *5 rats/group). Different superscript letters indicate a significant difference at *P* ≤ 0.05

The 30% feed-restricted group showed upregulation of the ghrelin gene in the stomach, hypothalamus, and testis tissues. In contrast, the 60% feed-restricted group exhibited significant upregulation of ghrelin transcription in all the examined organs, as shown in (Fig. 4).

### The Impact of Feed Restriction on Histopathological Analysis in Male Rats

**Fig. 5:**
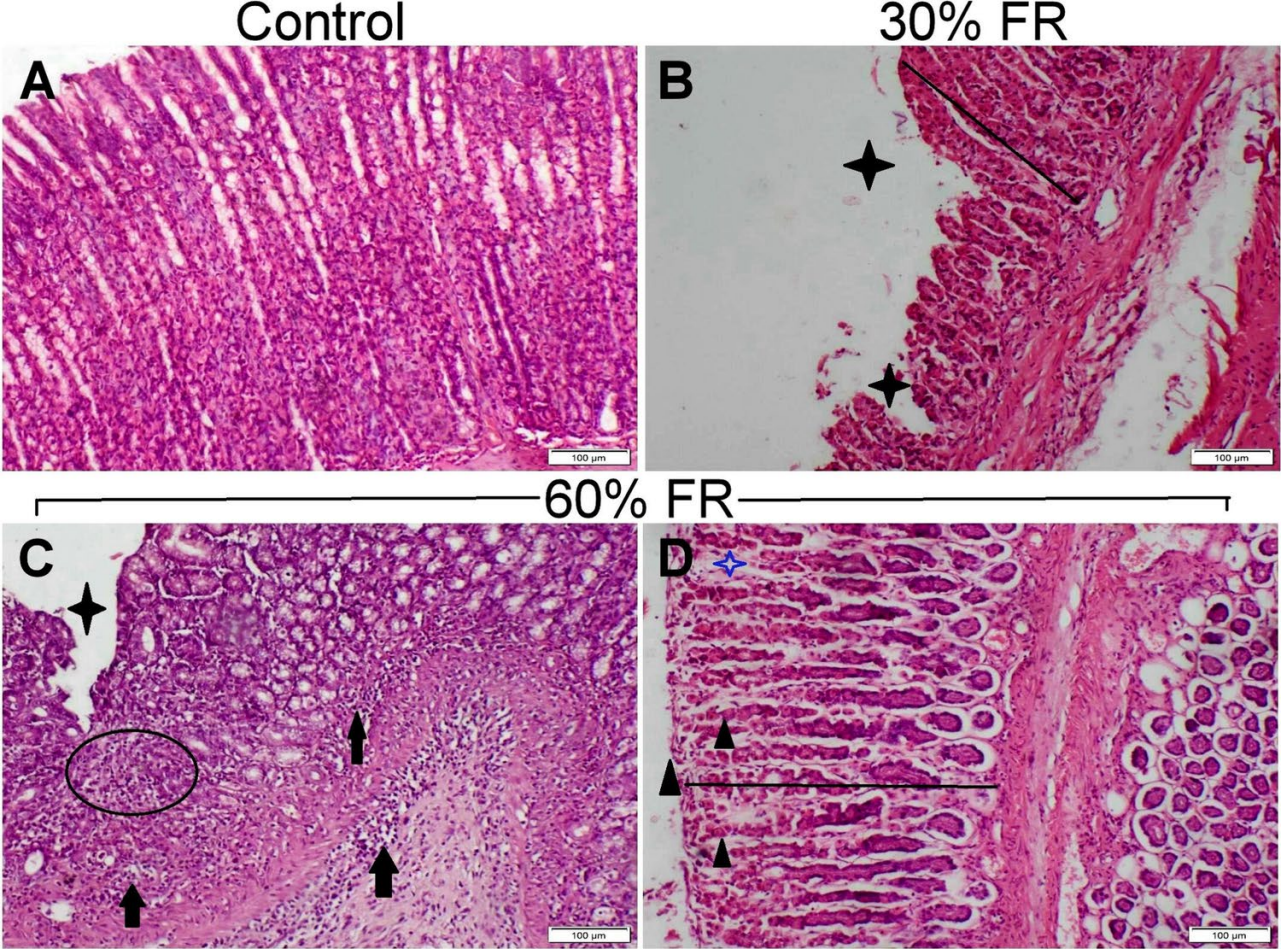
The impact of feed restriction on the histopathological analysis of the gastric fundus in male rats. Histopathological alterations of gastric fundus sections stained with H&E representing different experimental groups. **A** control group showed normal histological structure. **B** food restricted group 30% (30% FR) showed atrophy of gastric mucosa (line) with focal ulceration (star). **C-D** food restricted group 60% (60% FR) showed atrophy (line) and focal ulceration of gastric mucosa (star), inflammatory cells infiltration (arrow), loss of gastric glands (circle), necrosis and exfoliation of lining cells (triangle), fibrinous exudation (blue star). (scale bare = 100um Corresponding to magnification power × 10)

The gastric fundus of the control group exhibited a normal histological structure, including fundic glands, gastric pits, and lining cells (Fig. 5A). In contrast, the fundus of the 30% feed-restricted group displayed ulceration of the gastric pits in some sections, along with vascular congestion and desquamation of portions of the mucosal epithelium (Fig. 5B). The 60% feed-restricted group showed severe necrosis in both surface and glandular epithelial cells, accompanied by extensive infiltration of inflammatory cells (Fig. 5C). Additionally, a pseudomembrane consisting of necrotic cell debris and fibrin was observed covering the gastric mucosa (Fig. 5D).

**Fig. 6:**
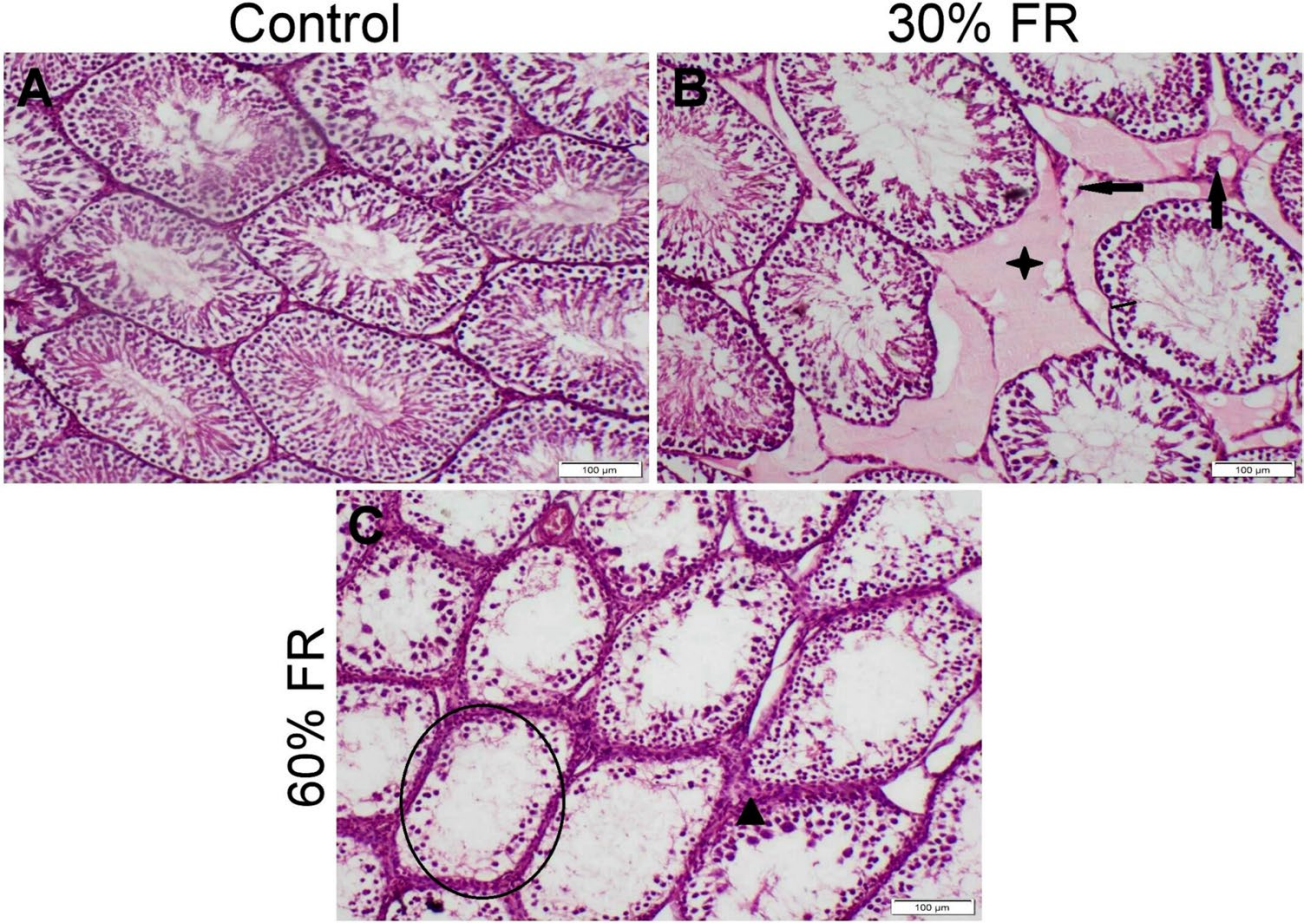
The impact of feed restriction on the histopathological analysis of the testis in male rats. Histopathological alterations of testicular sections stained with H&E representing different experimental groups. **A** control group showed normal histological structure. **B** food restricted group 30% (30% FR) showed decreased number of spermatogenic cells (line), Leydig cells degeneration (arrow), interstitial edema (star). **C** food restricted group 60% (60% FR) showed empty tubules lined with only spermatogonia (circle), hyperplasia of Leydig cells (triangle). (scale bare = 100um Corresponding to magnification power × 10)

The testis in the control group displayed a typical histological structure with homogenous seminiferous tubules lined by several spermatogenic cells, including spermatogonia, primary and secondary spermatocytes, spermatids, and spermatozoa, surrounded by scant interstitial tissue containing intact Leydig cells (Fig. 6A). In contrast, the 30% feed-restricted group exhibited interstitial edema and deterioration of spermatogenic cells (Fig. 6B). The 60% feed-restricted group displayed severe deterioration in spermatogenesis, with most seminiferous tubules showing edema and a complete loss of many spermatogenic cells, such as primary and secondary spermatocytes, spermatids, and spermatozoa. Hyperplasia of Leydig cells was also observed (Fig. 6C).

The results of histopathological grading indicated the highest scores in all parameters for the 60% feed-restricted group, followed by the 30% feed-restricted group (Table 3).

**Table 3:** Histopathologic scoring for the examined tissue sections

	Control	30% FR	60% FR
Gastric fundus lesion scoring			
Sloughing of gastric pits	**-**	** + + **	** + + + **
Atrophy of gastric mucosa	**-**	** + **	** + + **
Inflammatory cells	**-**	**-**	** + + + **
Damage of gastric glands	**-**	** + **	** + + **
Testicular lesion scoring			
Loss of spermatogonia	**-**	**-**	**-**
Loss of spermatocytes	**-**	** + **	** + + + **
Loss of spermatids	**-**	** + **	** + + + **
Loss of spermatozoa	**-**	** + + **	** + + + **
Spermatid giant cells	**-**	**-**	** + + **
Interstitial edema	**-**	** + + **	** + **
Leydig cell degeneration	**-**	** + + **	** + **
Leydig cell hyperplasia	**-**	**-**	** + + + **

(0) normal histology, (+) mild changes, (+ +) moderate changes, (+ + +) severe changes

## Discussion

The present study was carried out to explore the effects of feed restriction on the production and distribution of the ghrelin hormone in the body. Additionally, it analyzed the impact of ghrelin-induced feed restriction on reproductive hormones and mating behaviors in adult male rats. Our findings demonstrated that a 60% feed restriction led to a significant elevation in serum ghrelin and prolactin levels, with a concurrent decline in luteinizing hormone (LH), follicle-stimulating hormone (FSH), and testosterone concentrations. These hormonal alterations were less pronounced in the 30% feed-restricted group. The observed elevation in serum ghrelin concurs with previous reports, such as those by Tezenas du Montcel et al. (2023), who described a feed restriction-induced rise in ghrelin dependent on restriction duration and severity [26]. The elevation in serum ghrelin levels may be attributed to the interplay between ghrelin and signals regulating long-term energy balance, particularly insulin and leptin [27]. During states of energy deficit, including fasting or restricted caloric intake, the signaling of both insulin and leptin is diminished [28]. This reduction contributes to the noted elevation in serum ghrelin levels under such conditions [29].

The suppression of the hypothalamic-pituitary–gonadal (HPG) axis under caloric restriction is consistent with reduced LH and testosterone levels, which are known to impair Leydig cell function and spermatogenesis. Ghrelin has been shown to inhibit GnRH pulsatility, suppress LH secretion, and decrease testosterone production by downregulating Kiss1 expression in the hypothalamus. This decrease subsequently impacts androgen-dependent reproductive organs in male rats, leading to reduced organ weights [30] and compromised reproductive hormone profiles observed in our study, particularly in the 60% feed-restricted group. This result is in agreement with the findings of Abou Heif et al. (2010), who noted a significant decrease in male reproductive hormone secretion under conditions of feed restriction [31]. Similarly, Govic et al. (2008) reported that testosterone levels were equally diminished in adult male rats subjected to both 25% and 50% calorie restriction [32].

Feed restriction significantly reduced both testicular and final body weight, with the 60% feed-restricted group showing the most pronounced reduction. These findings align with the results reported by Rizzoto et al. (2019), who observed that adult male rats exposed to moderate and intense food restriction for eight weeks exhibited significant reductions in body and testicular weights under intense restriction, whereas moderate restriction produced results comparable to the control group [33]. Elevated ghrelin levels led to a decrease in luteinizing hormone and testosterone, which contributed to a reduction in total body mass and testicular weight [34, 35]. The observed reduction in the testis-to-body weight ratio in the 60% feed-restricted group reflects a significant compromise in reproductive organ development relative to somatic growth. This disproportionate decline suggests that under conditions of prolonged nutritional stress, the testes are particularly susceptible to atrophy, likely due to both systemic energy deficits and endocrine dysfunction.

Prolactin levels were significantly elevated under severe restriction, possibly due to ghrelin-induced activation of growth hormone secretagogue receptors in the hypothalamus and pituitary. This finding aligns with studies by Jones and Jernigan (2010) and Messini et al. (2011), who reported that ghrelin may elevate prolactin via dopamine receptor antagonism. Prolactin elevation under prolonged caloric restriction could reflect an adaptive response to metabolic stress and energy conservation [36, 37].

Feed restriction negatively influenced sexual behavior, as evidenced by increased mount latency and reduced mount frequency, contact attempts, and licking behaviors. MF and ML are considered an indicator of sexual motivation, libido, and potency [38]. The significant increase in ML and the significant reduction in MF observed in feed-restricted rats may suggest that elevated levels of the ghrelin hormone negatively affect sexual behavior. The hormones of HPG axis facilitate sexual behavior in mammals, while ghrelin exerts an inhibitory effect on the activity of this axis [39]. Thus, it is hypothesized that ghrelin could diminish sexual behavior through the inhibition of the HPG axis [15]. The reduced testosterone levels and the significant disparity in body weight between feed-restricted males and females may further contribute to the decline in mating behavior. Typically, successful mating requires the male to be heavier than the female, so reduced male body weight could impair sexual performance. Similarly, Bertoldi et al. (2011) reported that elevated plasma ghrelin levels during food restriction contribute to decreased receptivity in female mice, demonstrating ghrelin's ability to inhibit female sexual behavior [40].

Semen analysis revealed that both 30% and 60% feed restriction significantly reduced sperm count, motility, and viability while increasing abnormal sperm morphology. The severity of these effects was greater in the 60% restricted group. These findings are consistent with earlier studies in poultry and rodents, where caloric restriction suppressed gonadotropin secretion and spermatogenesis, thereby reducing semen quality and testicular function [41]. Additionally, Rizzoto et al. (2019) reported that feed restriction hindered sexual maturation in prepubertal male rats, with the extent of the delay being proportional to the severity of the restriction [33]. Caloric restriction, therefore, has detrimental effects on male reproductive health, including reduced sperm production, compromised sperm quality, and decreased testis size. These findings indicate that severe feed restriction (60%) significantly disrupts reproductive hormonal balance, impairs sexual behavior, and reduces semen quality, ultimately leading to functional infertility in male rats. The observed infertility in the 60% restricted group highlights the crucial role of adequate nutrition in maintaining male reproductive health.

The current results demonstrate a significant overexpression of ghrelin mRNA in the stomach, hypothalamus, and testis of feed-restricted groups. These data align with the observations of Ahmed et al. (2010), who suggested that the upregulation of ghrelin expression in gastric tissue may be linked to the transcription factor Krüppel-like factor 4 (KLF4) [29]. Research has shown that fasting increases ghrelin expression and raises KLF4 levels, suggesting a functional association between them.

Supporting the observed increase in hypothalamic ghrelin expression in feed-restricted animals, Unniappan et al. (2004) reported that starvation in goldfish triggers a rise in hypothalamic ghrelin mRNA levels [42]. Similarly, Amole and Unniappan (2009) demonstrated a notable elevation in preproghrelin mRNA in the brains of fasted zebrafish compared to those fed ad libitum, correlating this with elevated circulating ghrelin during fasting [43]. Insulin could be involved in this regulatory process, as it is downregulated during fasting and upregulated in obesity, where it inhibits ghrelin production and secretion from the stomach [44].

The increased ghrelin gene expression observed in the testis of feed-restricted groups suggests a compensatory mechanism that balances energy homeostasis and reproductive function [11]. This upregulation reflects a complex adaptive response designed to conserve energy during caloric restriction [45]. Under such conditions, the body enters a state of metabolic and systemic stress, triggering physiological adaptations that prioritize energy conservation over reproduction [46]. One key driver of this response is the activation AMP-activated protein kinase (AMPK) [47], which is stimulated by energy deficiency [48]. AMPK activation enhances ghrelin expression to support energy conservation [49]. Energy deficiency also activates stress response pathways, including the hypothalamic–pituitary–adrenal (HPA) axis, resulting in an elevated release of glucocorticoids [50, 51]. Glucocorticoids can influence the expression of genes related to metabolic regulation, including the gene encoding ghrelin, by interacting with glucocorticoid response elements (GREs) located in the promoter region of the ghrelin gene, thereby enhancing its transcription [51, 52].

The ghrelin-like effects of feed restriction have been shown to significantly impact the histopathological structure of organs such as the testes and stomach fundus. These structural changes may be linked to physiological alterations triggered by elevated ghrelin levels as a result of dietary restrictions. In the stomach fundus, feed restriction caused atrophy of the gastric mucosa and localized ulceration. These results are in agreement with those reported by Makovicky et al. (2014), who documented superficial erosions in the glandular mucosa of the stomach due to feed restriction [53]. Such dietary limitations ca n compromise gastric mucosal integrity, increasing the risk of conditions like gastric ulcers due to changes in mucosal thickness and digestive secretion balance [54]. In the testes, feed restriction led to notable changes in cellular structure, potentially affecting spermatogenesis and hormone production. There was a decrease in the number of spermatogenic cells and degeneration of Leydig cells. The inverse relationship between ghrelin and reproductive hormones suggests that increased ghrelin levels due to feed restriction may negatively influence testosterone and other reproductive hormones, potentially compromising fertility [15, 55]. These findings are in agreement with studies conducted by Martins et al. (2020), which demonstrated that feed restriction significantly affects the structural integrity of the testes, particularly concerning hormone levels and reproductive health [56]. Restricted feeding has been shown to cause degeneration of the seminiferous epithelium and a reduction in spermatid numbers. For example, studies indicate that caloric restriction leads to lower serum testosterone levels, which, in turn, impacts testis size and function [57]. In experiments with bulls, feed restriction during calfhood was associated with a diminished response of the HPG axis, leading to reduced levels of gonadotropins and testosterone [58]. Moreover, insufficient nutrition led to alterations in testicular histology, including reduced seminiferous tubule size and lower sperm production [59].

The findings of this study highlight the diagnostic and predictive significance of ghrelin in the context of reproductive health. Elevated serum ghrelin levels may serve as a valuable diagnostic marker for identifying states of malnutrition or energy deficiency, conditions that are known to adversely affect the onset of puberty and overall fertility. Furthermore, persistently high ghrelin concentrations in males may possess predictive value, indicating an increased risk of delayed pubertal development or future reproductive impairments. Thus, ghrelin could be utilized as a potential biomarker for the early detection of reproductive dysfunction in both experimental models and clinical assessments. we focused on evaluating the effect of elevated endogenous ghrelin levels, induced by feed restriction, on reproductive function in male rats. One limitation of the present study is that it primarily focused on evaluating the effects of elevated endogenous ghrelin levels, induced by feed restriction, on male reproductive function without direct mechanistic validation. Future studies should incorporate the use of ghrelin receptor antagonists to determine whether the observed reproductive alterations are specifically mediated by ghrelin signaling pathways.

## Conclusion

Feed restriction in male rats markedly elevates ghrelin levels, leading to a reduction in key reproductive hormones and an increase in prolactin levels. This hormonal imbalance negatively impacts reproductive function, semen quality, and the histopathological integrity of the stomach and testes. These findings emphasize the significant influence of dietary limitations on reproductive functions, highlighting the intricate interplay between nutrition and reproductive health in male physiology.

## Data Availability

All data will be available on request.
